# Supplementary material for: Clp protease and antisense RNA jointly regulate the global regulator CarD to mediate mycobacterial starvation response
Source: eLife. 2022 Jan 26;11:e73347. doi: 10.7554/eLife.73347 (PMC8820732; doi:10.7554/eLife.73347)
Supplement: Supplementary file 1. [file elife-73347-supp1.docx]

**Supplementary File 1 The top 20 up-regulated genes in the CarD^K125A^ mutant**

| **Gene^a^** | **Fold change^b^** | **Protein family^c^** | **Function^c^** |
| --- | --- | --- | --- |
| *Rv2623* | 36.03 | [pfam00582](http://pfam.xfam.org/family/pf00582) | Universal stress protein |
| *Rv2624c* | 25.19 | [pfam00582](http://pfam.xfam.org/family/pf00582) | Universal stress protein |
| *Rv3129* | 19.46 | [pfam12900](http://pfam.xfam.org/family/pf12900) | Pyridoxamine 5'-phosphate oxidase family protein |
| *Rv2626c* | 17.49 | [pfam00571](http://pfam.xfam.org/family/pf00571) | Hypoxic response protein 1, CBS domain pair |
| *Rv3128c* | 15.14 | N/A | DDE-type integrase/transposase/recombinase |
| *Rv2030c* | 14.41 | [pfam05139](http://pfam.xfam.org/family/pf05139) | Erythromycin esterase family protein |
| *Rv2007c* | 11.51 | [COG1146](https://ftp.ncbi.nih.gov/pub/COG/COG2014/static/byCOG/COG1146.html) | Ferredoxin FdxA |
| *Rv2029c* | 11.42 | [COG1105](https://ftp.ncbi.nih.gov/pub/COG/COG2014/static/byCOG/COG1105.html) | 6-Phosphofructokinase PfkB |
| *Rv2625c* | 11.39 | [COG1994](https://ftp.ncbi.nih.gov/pub/COG/COG2014/static/byCOG/COG1994.html) | Putative zinc metalloprotease Rip3 |
| *Rv2627c* | 10.95 | N/A | Alpha/beta hydrolase |
| *Rv0022c* | 9.31 | [pfam02467](http://pfam.xfam.org/family/pf02467) | Transcriptional regulator WhiB5 |
| *Rv2031c* | 7.21 | [pfam00011](http://pfam.xfam.org/family/pf00011) | Heat shock protein HspX |
| *Rv1736c* | 6.74 | [COG5013](https://ftp.ncbi.nih.gov/pub/COG/COG2014/static/byCOG/COG5013.html) | Nitrate reductase NarX |
| *Rv1738* | 5.89 | [pfam08962](http://pfam.xfam.org/family/pf08962) | Bacterial hibernation-promoting factor (PDB: 4wpy) |
| *Rv2628* | 5.66 | N/A | Hypothetical protein |
| *Rv1997* | 5.56 | [COG0474](https://ftp.ncbi.nih.gov/pub/COG/COG2014/static/byCOG/COG0474.html) | Cation-transporting ATPase CtpF |
| *Rv2028c* | 5.52 | [pfam00582](http://pfam.xfam.org/family/pf00582) | Universal stress protein |
| *Rv1130* | 5.06 | [COG2079](https://ftp.ncbi.nih.gov/pub/COG/COG2014/static/byCOG/COG2079.html) | 2-methylcitrate dehydratase PrpD |
| *Rv1733c* | 4.87 | N/A | Transmembrane protein |
| *Rv1737c* | 4.62 | [COG2223](https://ftp.ncbi.nih.gov/pub/COG/COG2014/static/byCOG/COG2223.html) | Nitrate/nitrite transporter NarK2 |

**^a^**- The underlined genes belong to the dormancy regulon identified previously.

**^b^**- Change in gene expression in the CarD^K125A^ mutant compared to the wild-type strain, based on the previously published RNA-seq data (GEO accession number: GSE131043).

**^c^** - Protein family assignments in Pfam and COG databases, where available. Functional assignments are from GenBank and/or RefSeq.
