# Supplementary material for: Clp protease and antisense RNA jointly regulate the global regulator CarD to mediate mycobacterial starvation response
Source: eLife. 2022 Jan 26;11:e73347. doi: 10.7554/eLife.73347 (PMC8820732; doi:10.7554/eLife.73347)
Supplement: Supplementary file 2. [file elife-73347-supp2.docx]

**Supplementary File 2 Strains used in this study**

| **Strain** | **Characteristics used in this work** | **Source** |
| --- | --- | --- |
| mc^2^155 | Wild-type strain of *M. smegmatis* mc^2^155, abbreviated as Ms | Lab stock |
| BCG | Wild-type strain of *M. bovis* BCG | Lab stock |
| H37Ra | Wild-type strain of *M. tuberculosis* H37Ra | Lab stock |
| Ms/pMV261 | mc^2^155 with pMV261 empty vector | This study |
| *ascarD*_OE_ | *ascarD* overexpression strain | This study |
| Ms/pRH2521 | mc^2^155 with pRH2521 empty vector | This study |
| *ascarD*_KD_ | *ascarD* knock-down strain | This study |
| *carD*_OE_ | *carD* overexpression strain | This study |
| Ms/pRH2502*-clpP2* | mc^2^155 strain integrated with pRH2502*-clpP2* plasmid | This study |
| *clpP2*CM | *clpP2* conditional mutant | This study |
| AAAS_del | CarD C-terminal “AAAS” motif deletion mutant | This study |
| Ms/pRH2502*-clpC1*-His | used for the ClpC1 protein level determination | This study |
| Δ*sigF* | mc^2^155 with *sigF* knocked out | This study |
| Ms/pMV261-P*ascarD*-*lacZ* | used for the promoter activity determination of *ascarD* in mc^2^155 strain | This study |
| Δ*sigF*/pMV261-P*ascarD*-*lacZ* | used for the promoter activity determination of *ascarD* in Δ*sigF* strain | This study |
| Ms/P_PUCP_ | used for the promoter activity determination of P_PUCP_ in mc^2^155 strain | This study |
| Ms/P_PUCP*_ | used for the promoter activity determination of P_PUCP*_ in mc^2^155 strain | This study |
| AsM | *ascarD* promoter mutant, with the -10 motif changed from “GGGTA” to “**C**GG**C**A” | This study |
| AsM/AAAS_del | Double mutant of the *ascarD* promoter and CarD “AAAS” motif | This study |
