# Supplementary material for: Clp protease and antisense RNA jointly regulate the global regulator CarD to mediate mycobacterial starvation response
Source: eLife. 2022 Jan 26;11:e73347. doi: 10.7554/eLife.73347 (PMC8820732; doi:10.7554/eLife.73347)
Supplement: Supplementary file 3. [file elife-73347-supp3.docx]

**Supplementary File 3 Oligonucleotides used in this study**

| **Primer name** | **Use in this work** | **Sequence 5′–3′** |
| --- | --- | --- |
| RT-*ascarD*-F | Forward primer for AscarD detection by RT-PCR | TCTCGGCCTTGGCGTCATC |
| RT-*ascarD*-R | Reverse primer for AscarD detection by RT-PCR | GTCGCGTCGCTACAAGGC |
| RT-*carD*-F | Forward primer for *carD* transcript detection by RT-PCR | TCGTCTATCCACACCACGGTGC |
| RT-*carD*-R | Reverse primer for *carD* transcript detection by RT-PCR | TCGGCTCCTCGGTGTGCG |
| RT-*ispD*-F | Forward primer for *ispD* transcript detection by RT-PCR | CAGACGCCTCAGGGTTTCCA |
| RT-*ispD*-R | Reverse primer for *ispD* transcript detection by RT-PCR | ATCTTGAATGCCAGCGGGTC |
| RT-*sigA*-F | Forward primer for *sigA* detection by RT-PCR | CGTTCCTCGACCTCATCCAG |
| RT-*sigA*-R | Reverse primer for *sigA* transcript detection by RT-PCR | TGATCACCTCGACCATGTGC |
| *carD*_OE_-F | Forward primer for *carD* overexpression | CCGGAATTCATTTTTAAGGTCGGAGACACCGTC |
| *carD*_OE_-R | Reverse primer for *carD* overexpression | ATAGATATCGGACGCGGCGGCCAAAACCTCGT |
| *clpP2*-F | Forward primer for *clpP2* amplification | gcgaaggagatatacatatgATGAGCAACATaCATCCGTC |
| *clpP2*-R | Reverse primer for *clpP2* amplification | gttccgagtttgttaattaaACCCGTTGTCGTGTCAGA |
| *clpP2*-crRNA-F | Forward primer of *clpP2*-crRNA | ATATCCGTCACTGGACGCCCGGCTGCAA |
| *clpP2*-crRNA-R | Reverse primer of *clpP2*-crRNA | AGCTTTGCAGCCGGGCGTCCAGTGACGGATATCT |
| *clpP2*-lag | 59 nt lag sequence (targeting the lagging strand of DNA replication), used for *clpP2* mutation | GACGGCAGGATGTAGCGGGCCTGCGGCTttaCGGATGAATGTTGCTCATTTGTCTAGTC |
| AAAS-crRNA-F | Forward primer of AAAS-crRNA | ATTGGCCGCCGCGTCCTGATCTGTTCGA |
| AAAS-crRNA-R | Reverse primer of AAAS-crRNA | AGCTTCGAACAGATCAGGACGCGGCGGCCAATCT |
|  |  |  |
| AAAS-lag | 79 nt lag sequence, used for CarD AAAS motif deletion | ACGGCAACCGTCGCCACCGTTTACGTCCCGAACAGATCACAAAACCTCGTCGAGGATGGTCTCGGCCTTGGCGTCATCG |
| *clpC1*-F | Forward primer for *clpC1*-His construction | atctagacatcatcggtaccCGCAACGGACACAATGTGTCGA |
| *clpC1*-R | Reverse primer for *clpC1*-His construction | gttccgagtttgttaattagtggtggtggtggtggtgCTCCGTGCCTGCGGTCGG |
| 5’-RACE-*ascarD*-F | Forward primer for AscarD TSS identification | GACCACGCGTATCGATGTCGACTTTTTTTTTTTTTTTTV |
| 5’-RACE-*ascarD*-R | Reverse primer for AscarD TSS identification | CCCGCGCTCACCGACGAGTCGAAAC |
| *sigF*-crRNA-F | Forward primer of *sigF*-crRNA | ATCGCGACAACAGCTGGTCGGTGAAGGA |
| *sigF*-crRNA-R | Reverse primer of *sigF*-crRNA | AGCTTCCTTCACCGACCAGCTGTTGTCGCGATCT |
| *sigF*-lag | 59 nt lag sequence, used for *sigF* mutation | GGAGTTCCTTGAGCCGGCGAGGCACCTTCAGTGCCTGCGGACCTCGCCCATGATCGTGG |
|  |  |  |
| *ascarD*-sgRNA-F | Forward primer of *ascarD*-sgRNA | GGGAGCGCTCGCTCTCGAAGCGGC |
| *ascarD*-sgRNA-R | Reverse primer of *ascarD*-sgRNA | AAACGCCGCTTCGAGAGCGAGCGC |
| *ascarD*_OE_-F | Forward primer for *ascarD* overexpression | GCTCTAGACCAGGCACAGCAGACGACAC |
| *ascarD*_OE_-R | Reverse primer for *ascarD* overexpression | CCCAAGCTTTGATCTGGGCCCGTGTTAGG |
| P*ascarD*-*lacZ* -F | Forward primer for the construction of *ascarD* promoter reporting plasmid | CGGTACCAGATCTTTAAATCTAGACCAGGCACAGCAGACGACACGG |
| P*ascarD*-*lacZ* -R | Reverse primer for the construction of *ascarD* promoter reporting plasmid | AAACGACGGGATCCATTGAAGCTTCCGGGTGGTGGCTGCGCTGA |
| PUCP-F | Forward primer for PUCP construction | CGGTACCAGATCTTTAAATCTAGATGGCGATCATCAGGGGAATGT |
| PUCP-R | Reverse primer for PUCP construction | AAACGACGGGATCCATTGAAGCTTGGATGGTCTCGGCCTTGGCG |
| AsM-crRNA-F | Forward primer of *ascarD*-crRNA | ATCGAGCACCGCGCCGTTGGCGTCGACA |
| AsM-crRNA-R | Reverse primer of *ascarD*-crRNA | AGCTTGTCGACGCCAACGGCGCGGTGCTCGATCT |
| AsM-upstream-F | Forward primer of the *ascarD* upstream | GTCGAATTCCAGGCACAGCA |
|  |  |  |
| AsM-upstream-R | Reverse primer of the *ascarD* upstream | TACGCCCGAGCGGGCCGGTCTGCGCGCTGTCCAG |
| AsM-downstream-F | Forward primer of the *ascarD* downstream | CCGGCCCGCTCGGGCGTACCGAGCACCGCGCCGTTGGCGTCGACAGCCTTT |
| AsM-downstream-R | Reverse primer of the *ascarD* downstream | GAAAGCATTCGTGACACTGGGG |
