# Supplementary figures and images for: Clp protease and antisense RNA jointly regulate the global regulator CarD to mediate mycobacterial starvation response
Source: eLife. 2022 Jan 26;11:e73347. doi: 10.7554/eLife.73347 (PMC8820732; doi:10.7554/eLife.73347)

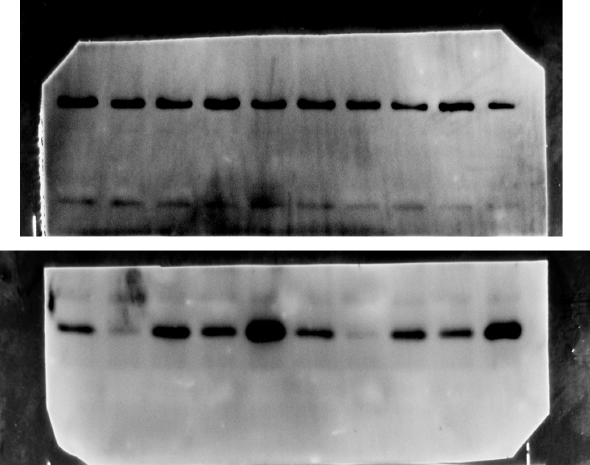

Supplement: Source data 1. [file elife-73347-supp4.zip › Raw_image/Raw image for Figure 1B-1.tif]

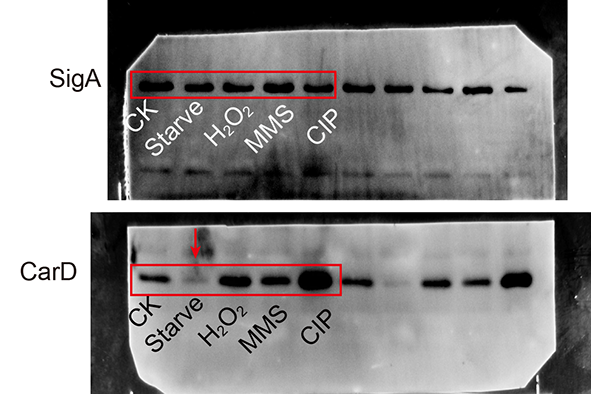

Supplement: Source data 1. [file elife-73347-supp4.zip › Raw_image/Raw image for Figure 1B-2.tif]

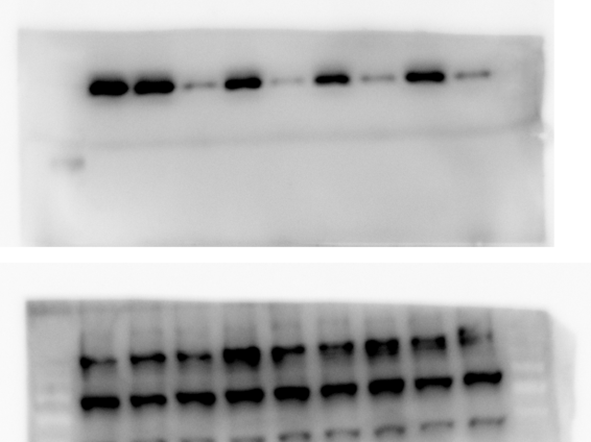

Supplement: Source data 1. [file elife-73347-supp4.zip › Raw_image/Raw image for Figure 1C-1.tif]

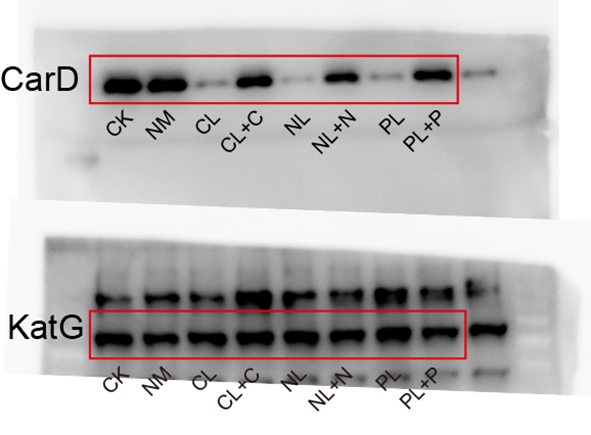

Supplement: Source data 1. [file elife-73347-supp4.zip › Raw_image/Raw image for Figure 1C-2.tif]

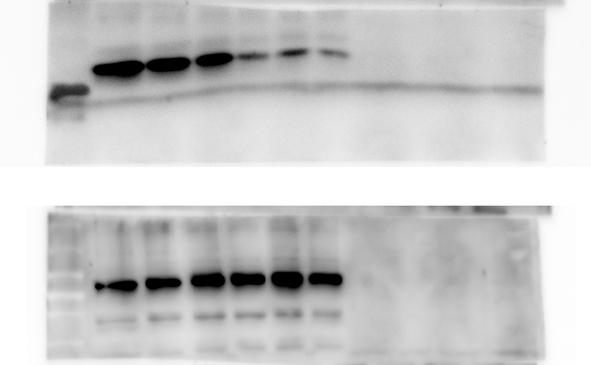

Supplement: Source data 1. [file elife-73347-supp4.zip › Raw_image/Raw image for Figure 1D-1.tif]

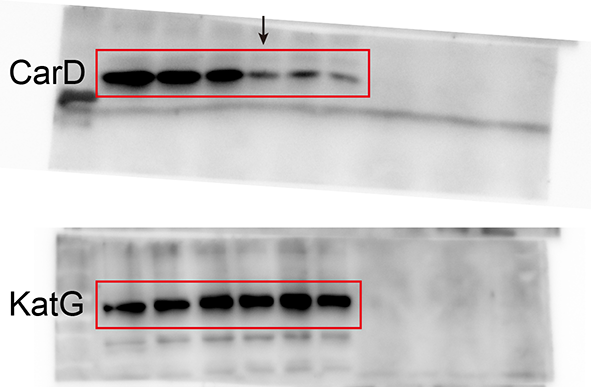

Supplement: Source data 1. [file elife-73347-supp4.zip › Raw_image/Raw image for Figure 1D-2.tif]

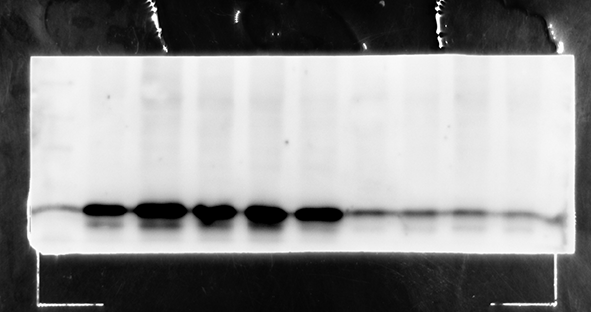

Supplement: Source data 1. [file elife-73347-supp4.zip › Raw_image/Raw image for Figure 1E-1.tif]

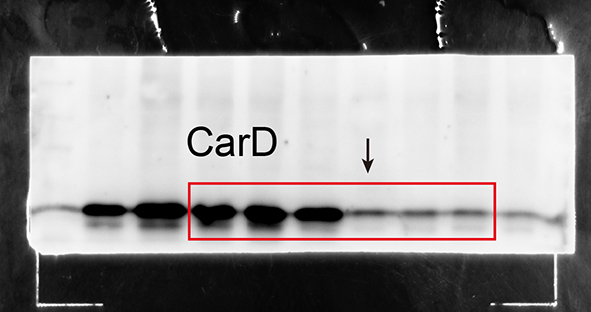

Supplement: Source data 1. [file elife-73347-supp4.zip › Raw_image/Raw image for Figure 1E-2.tif]

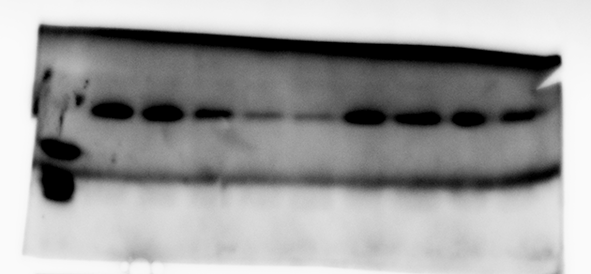

Supplement: Source data 1. [file elife-73347-supp4.zip › Raw_image/Raw image for Figure 1F-1.tif]

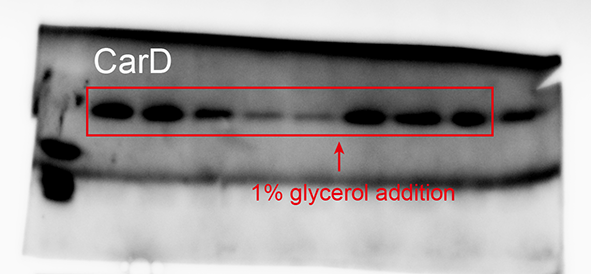

Supplement: Source data 1. [file elife-73347-supp4.zip › Raw_image/Raw image for Figure 1F-2.tif]

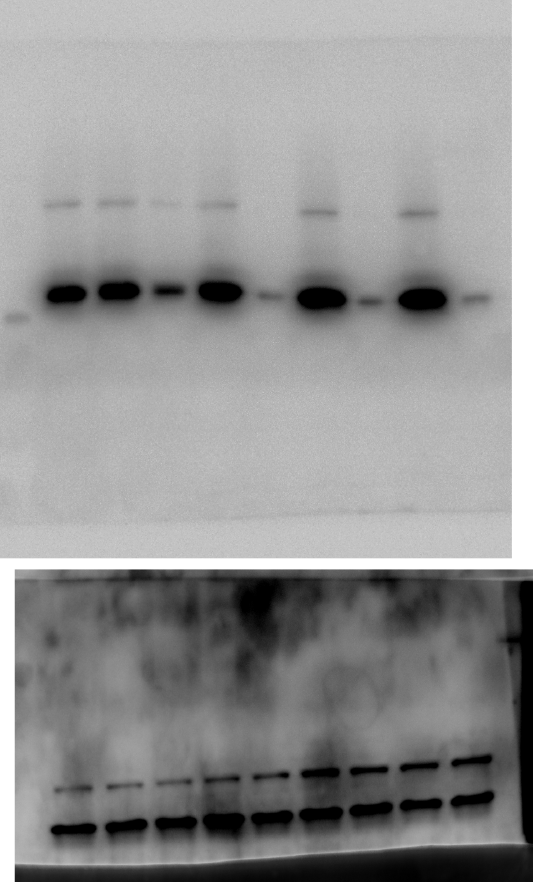

Supplement: Source data 1. [file elife-73347-supp4.zip › Raw_image/Raw image for Figure 2A-1.tif]

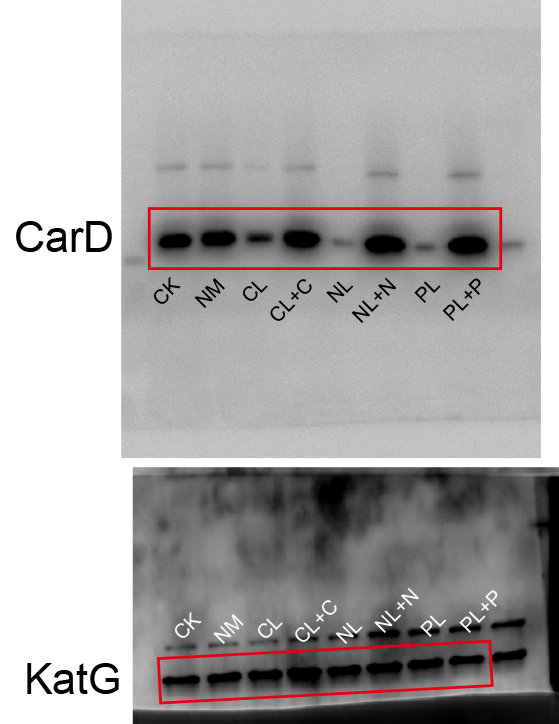

Supplement: Source data 1. [file elife-73347-supp4.zip › Raw_image/Raw image for Figure 2A-2.tif]

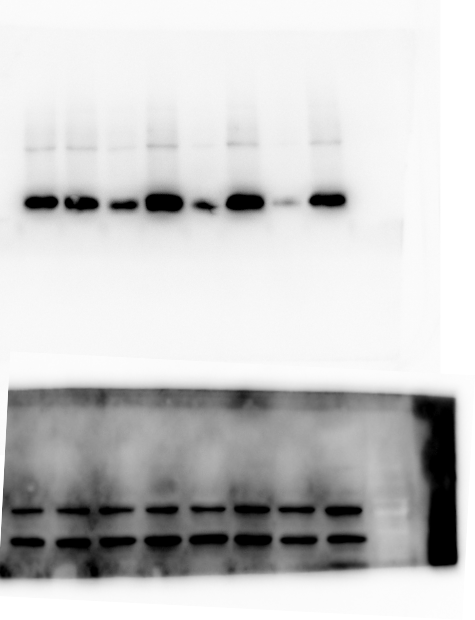

Supplement: Source data 1. [file elife-73347-supp4.zip › Raw_image/Raw image for Figure 2B-1.tif]

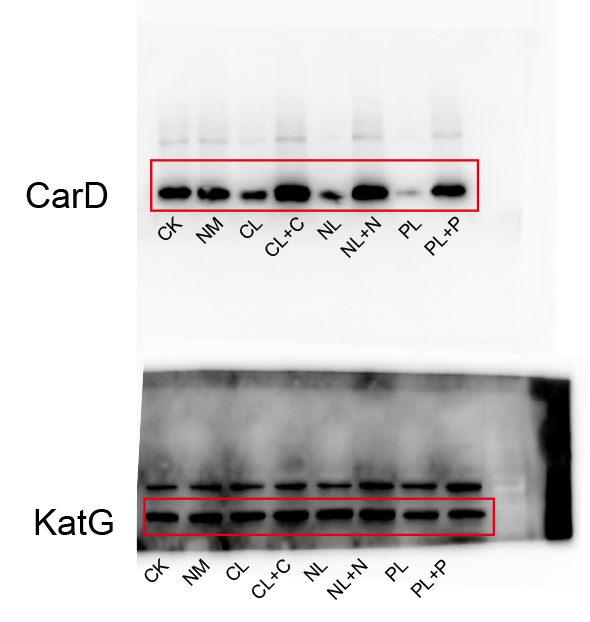

Supplement: Source data 1. [file elife-73347-supp4.zip › Raw_image/Raw image for Figure 2B-2.tif]

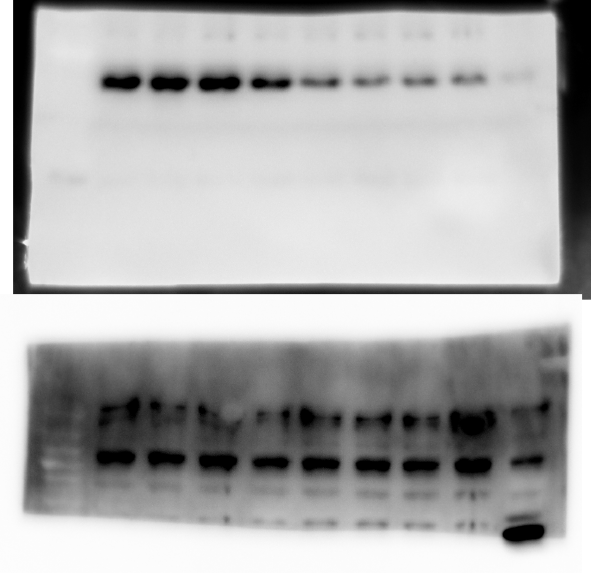

Supplement: Source data 1. [file elife-73347-supp4.zip › Raw_image/Raw image for Figure 2C-1.tif]

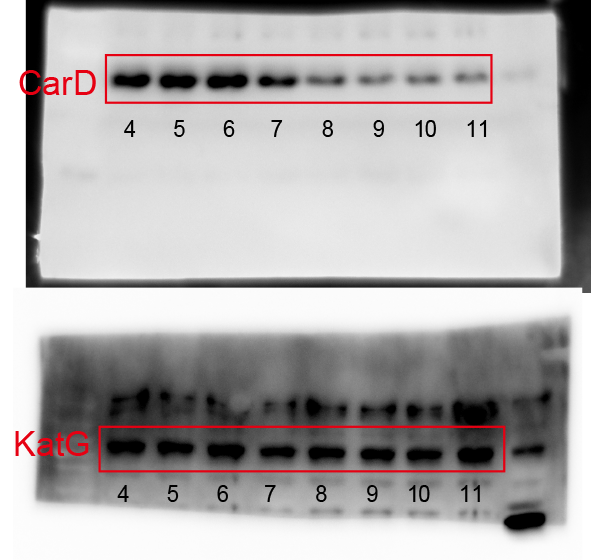

Supplement: Source data 1. [file elife-73347-supp4.zip › Raw_image/Raw image for Figure 2C-2.tif]

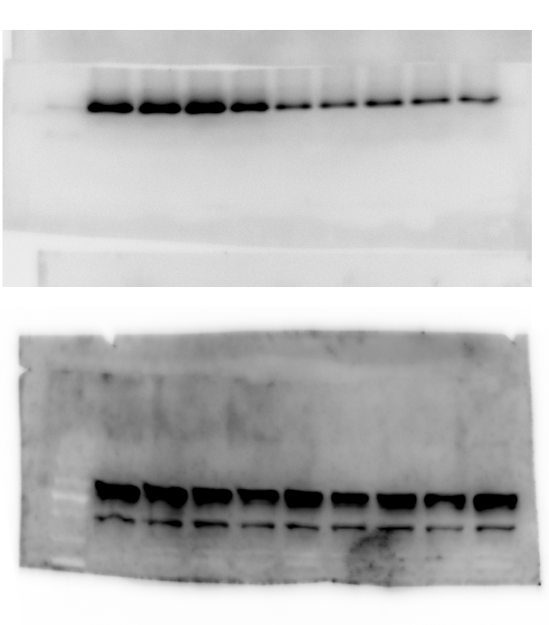

Supplement: Source data 1. [file elife-73347-supp4.zip › Raw_image/Raw image for Figure 2D-1.tif]

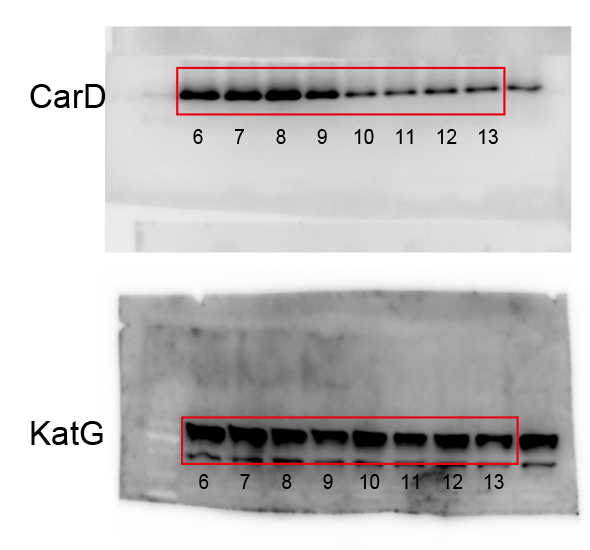

Supplement: Source data 1. [file elife-73347-supp4.zip › Raw_image/Raw image for Figure 2D-2.tif]

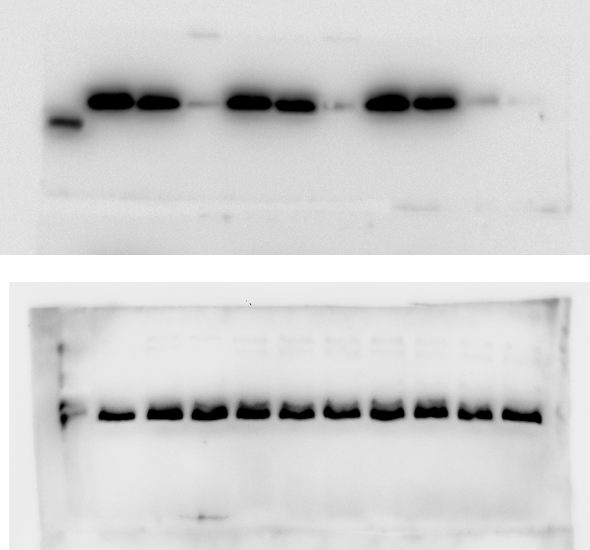

Supplement: Source data 1. [file elife-73347-supp4.zip › Raw_image/Raw image for Figure 2E-1.tif]

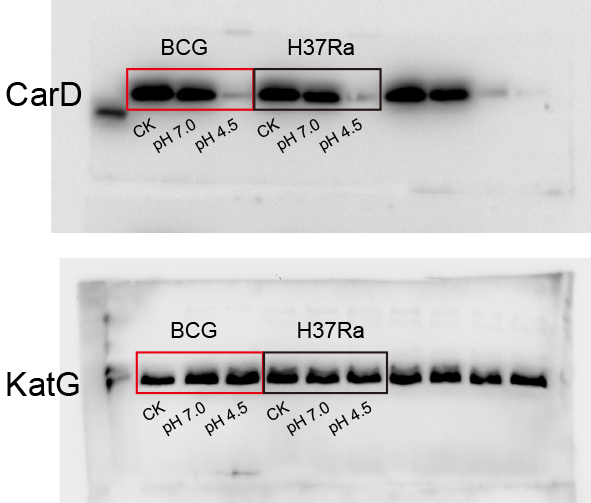

Supplement: Source data 1. [file elife-73347-supp4.zip › Raw_image/Raw image for Figure 2E-2.tif]

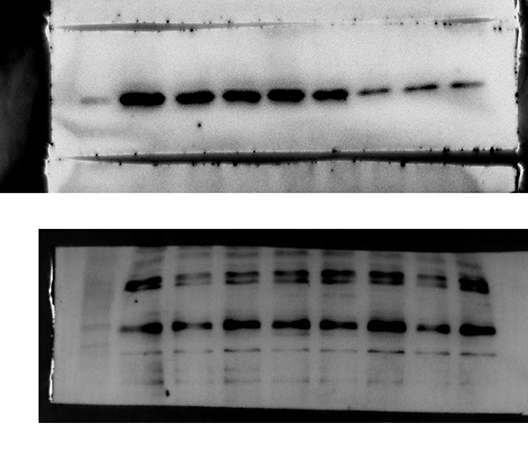

Supplement: Source data 1. [file elife-73347-supp4.zip › Raw_image/Raw image for Figure 3A-1.tif]

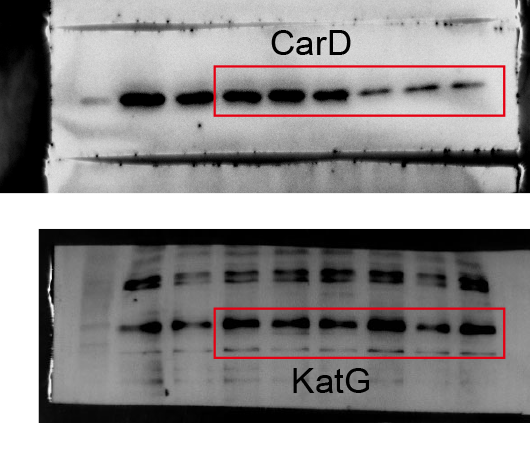

Supplement: Source data 1. [file elife-73347-supp4.zip › Raw_image/Raw image for Figure 3A-2.tif]

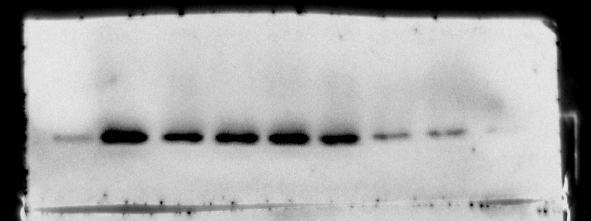

Supplement: Source data 1. [file elife-73347-supp4.zip › Raw_image/Raw image for Figure 3B-1.tif]

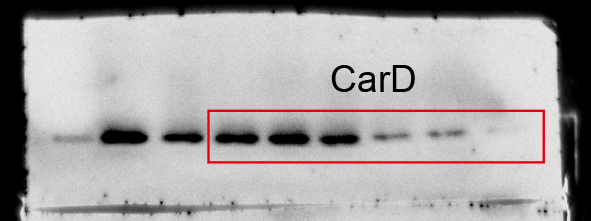

Supplement: Source data 1. [file elife-73347-supp4.zip › Raw_image/Raw image for Figure 3B-2.tif]

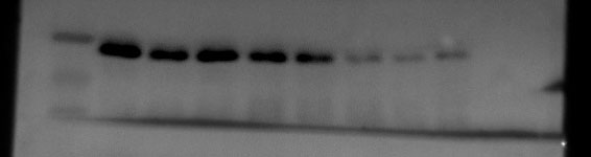

Supplement: Source data 1. [file elife-73347-supp4.zip › Raw_image/Raw image for Figure 3C-1.tif]

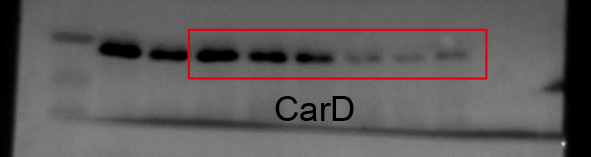

Supplement: Source data 1. [file elife-73347-supp4.zip › Raw_image/Raw image for Figure 3C-2.tif]

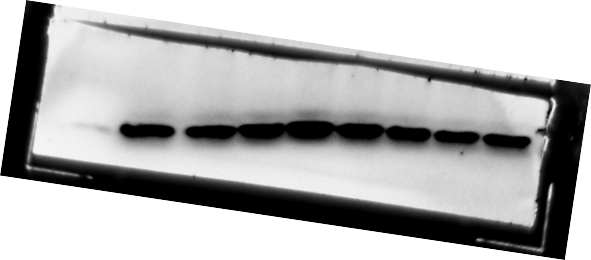

Supplement: Source data 1. [file elife-73347-supp4.zip › Raw_image/Raw image for Figure 3D-1.tif]

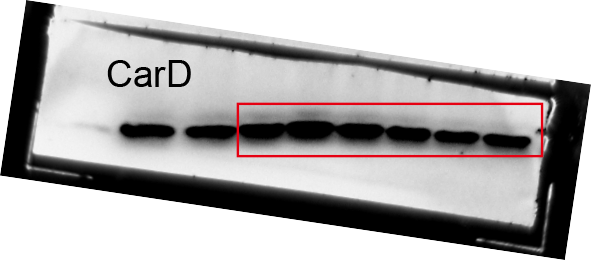

Supplement: Source data 1. [file elife-73347-supp4.zip › Raw_image/Raw image for Figure 3D-2.tif]

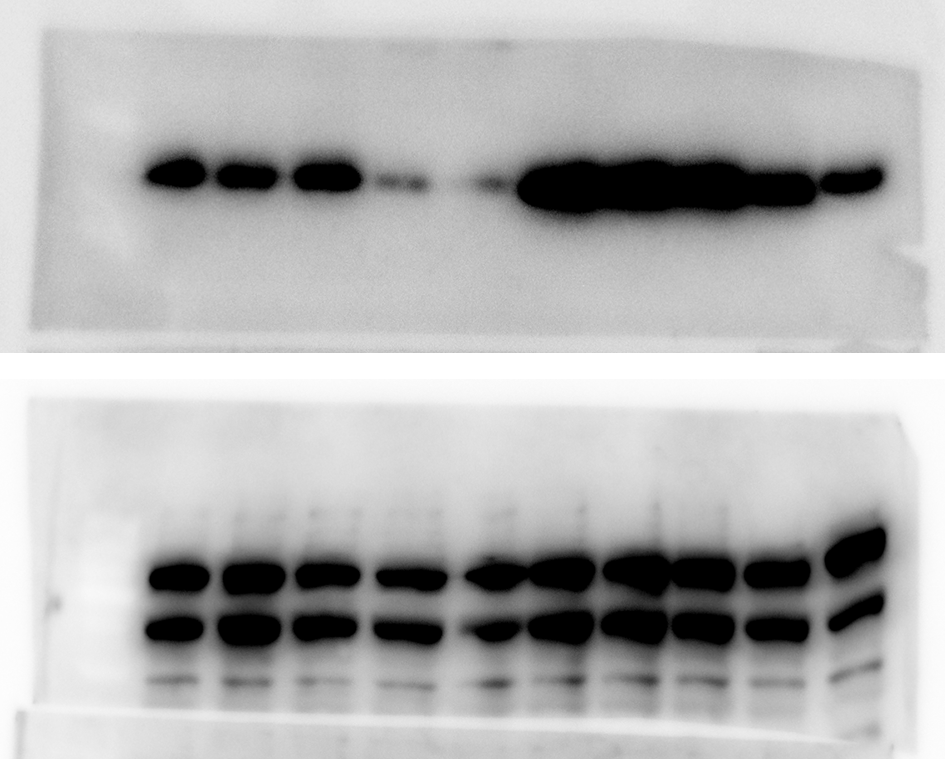

Supplement: Source data 1. [file elife-73347-supp4.zip › Raw_image/Raw image for Figure 3F-1.tif]

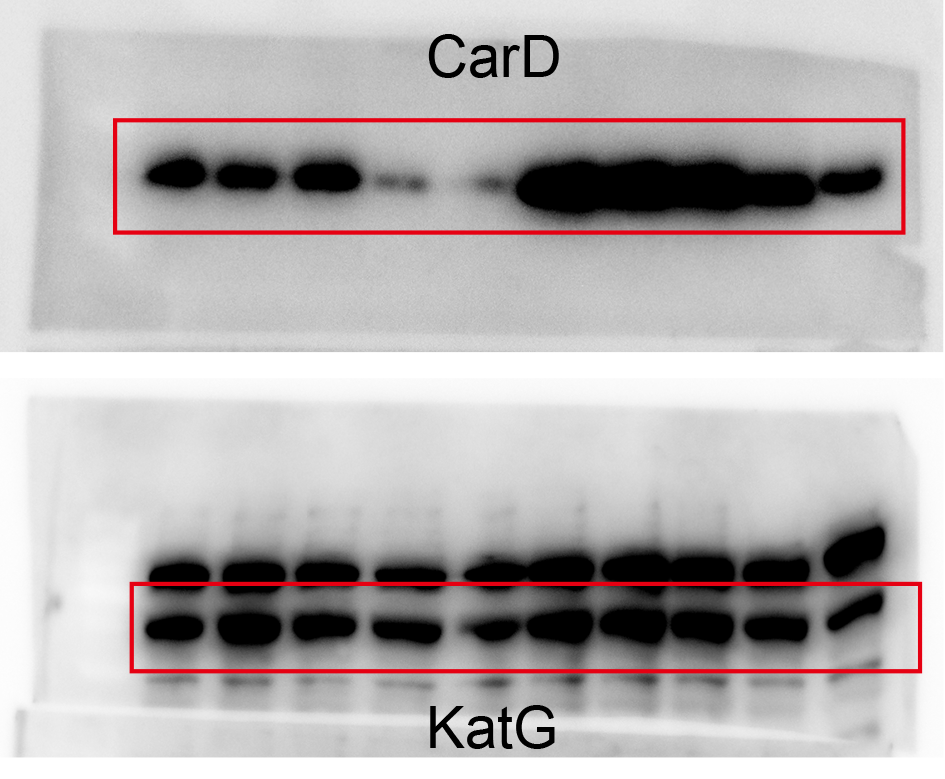

Supplement: Source data 1. [file elife-73347-supp4.zip › Raw_image/Raw image for Figure 3F-2.tif]

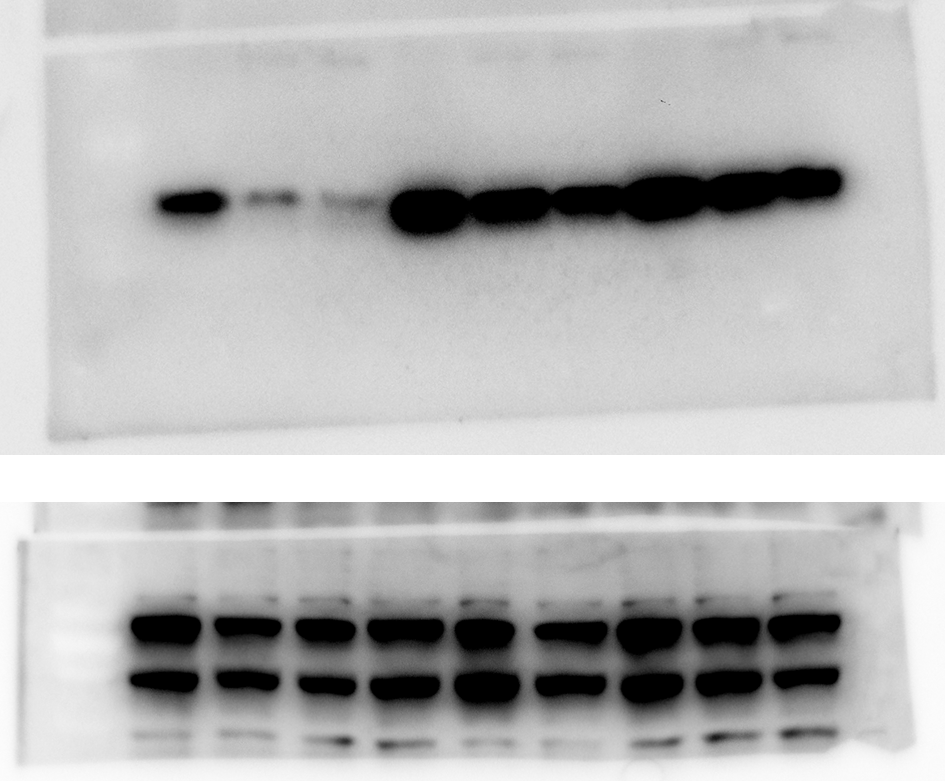

Supplement: Source data 1. [file elife-73347-supp4.zip › Raw_image/Raw image for Figure 3G-1.tif]

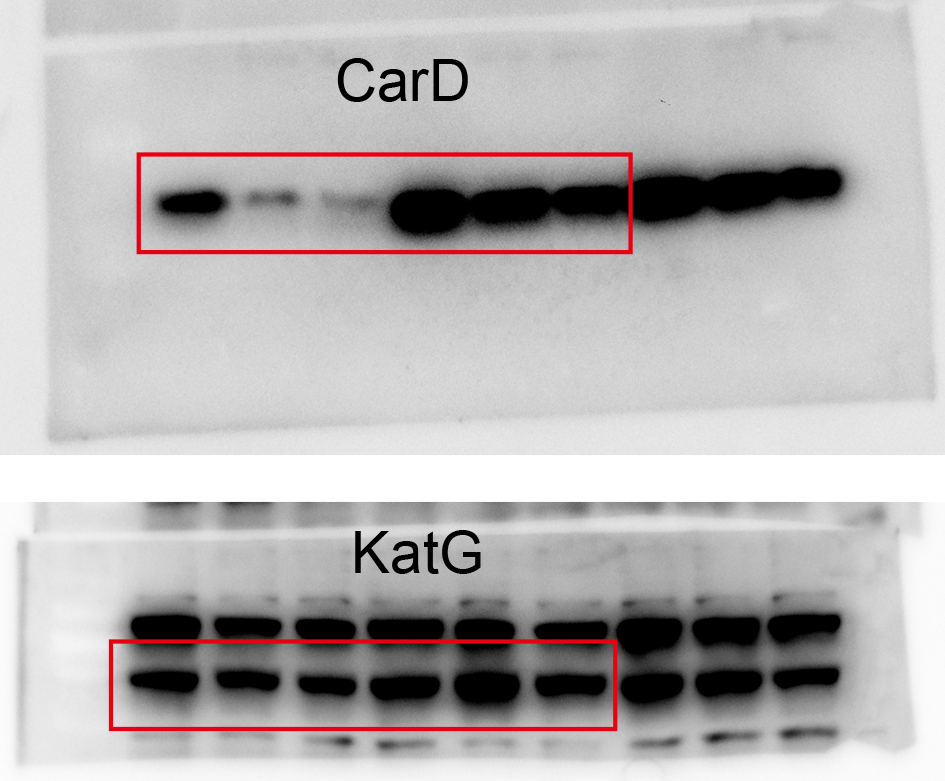

Supplement: Source data 1. [file elife-73347-supp4.zip › Raw_image/Raw image for Figure 3G-2.tif]

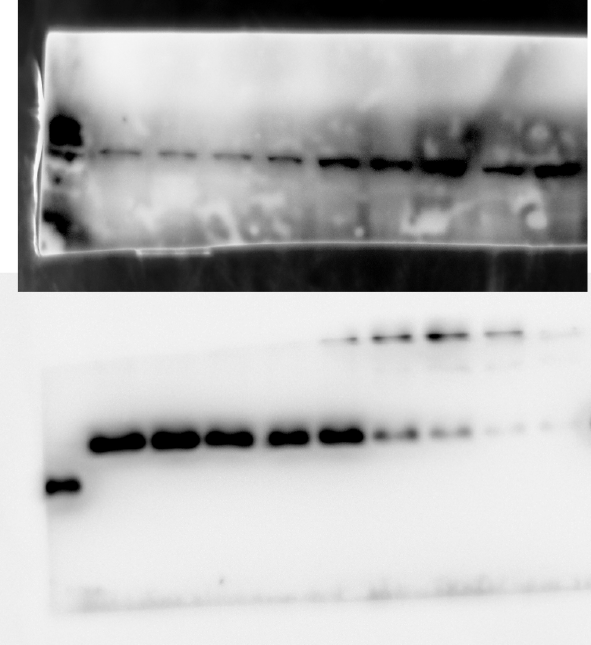

Supplement: Source data 1. [file elife-73347-supp4.zip › Raw_image/Raw image for Figure 3I-1.tif]

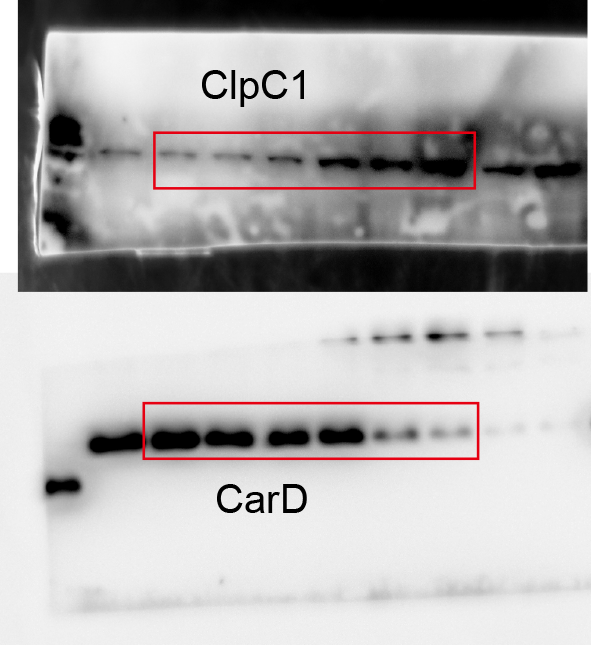

Supplement: Source data 1. [file elife-73347-supp4.zip › Raw_image/Raw image for Figure 3I-2.tif]

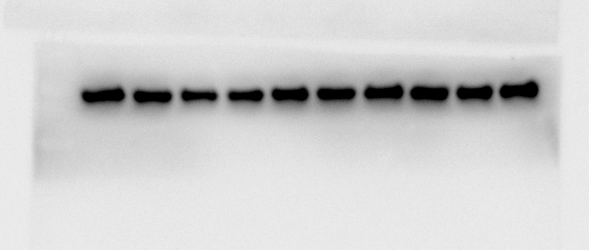

Supplement: Source data 1. [file elife-73347-supp4.zip › Raw_image/Raw image for Figure 3¿Cfigure supplement 2C-1.tif]

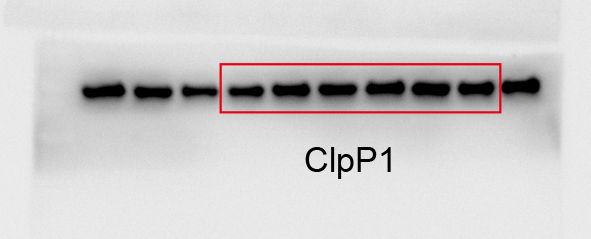

Supplement: Source data 1. [file elife-73347-supp4.zip › Raw_image/Raw image for Figure 3¿Cfigure supplement 2C-2.tif]

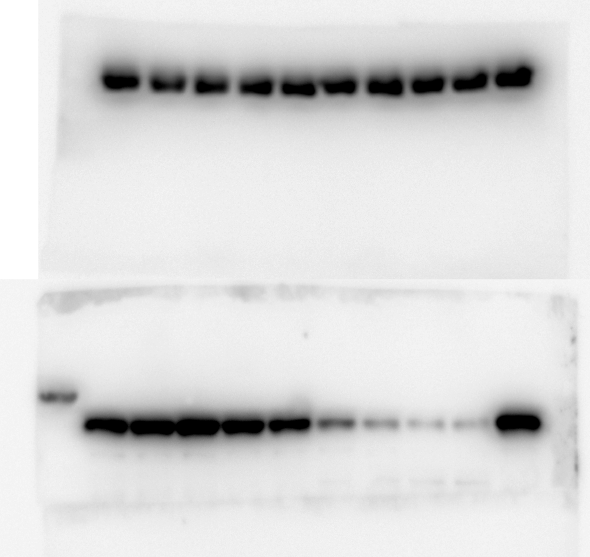

Supplement: Source data 1. [file elife-73347-supp4.zip › Raw_image/Raw image for Figure 3¿Cfigure supplement 2D-1.tif]

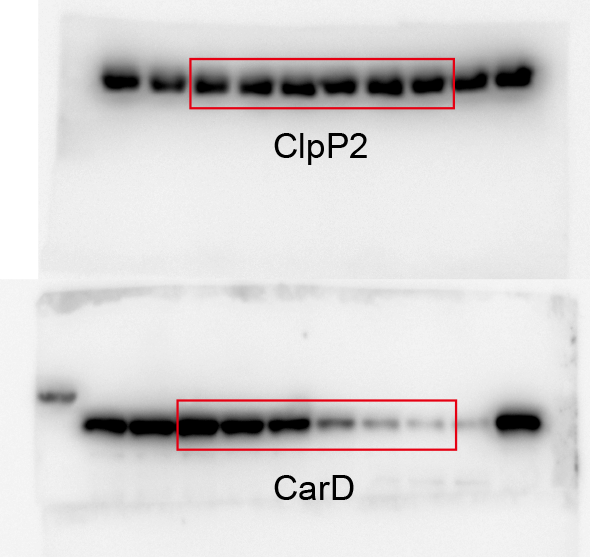

Supplement: Source data 1. [file elife-73347-supp4.zip › Raw_image/Raw image for Figure 3¿Cfigure supplement 2D-2.tif]

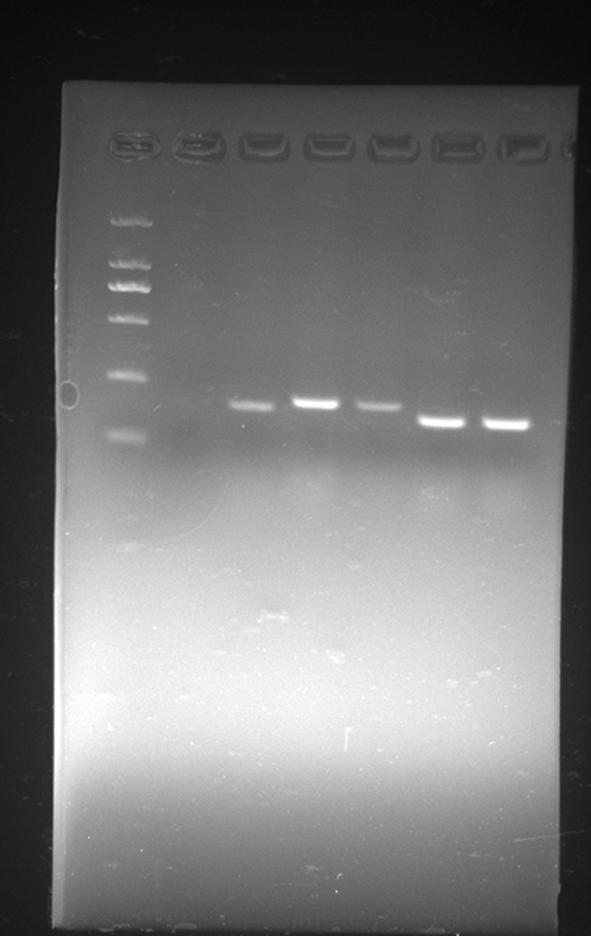

Supplement: Source data 1. [file elife-73347-supp4.zip › Raw_image/Raw image for Figure 4¿Cfigure supplement 1B-1.tif]

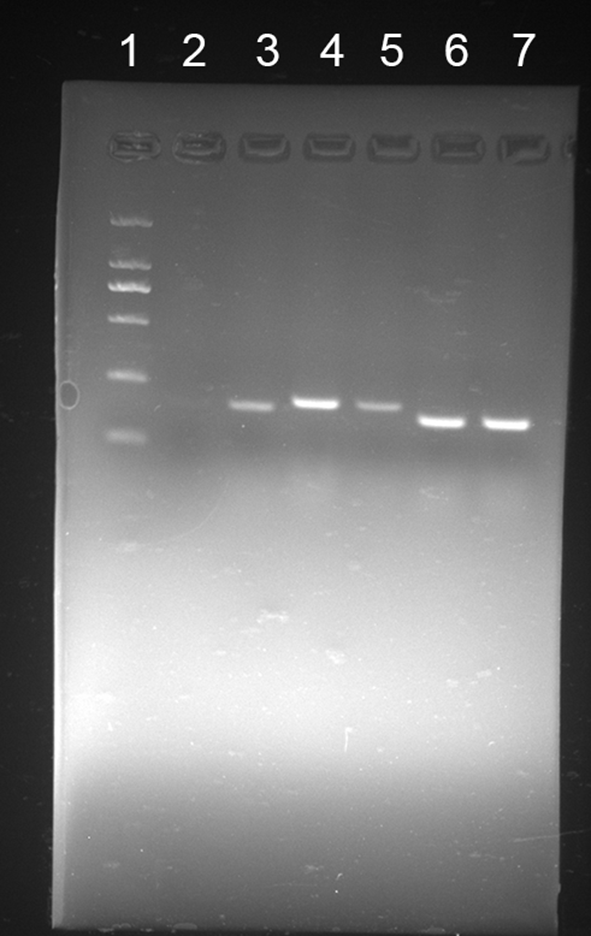

Supplement: Source data 1. [file elife-73347-supp4.zip › Raw_image/Raw image for Figure 4¿Cfigure supplement 1B-2.tif]

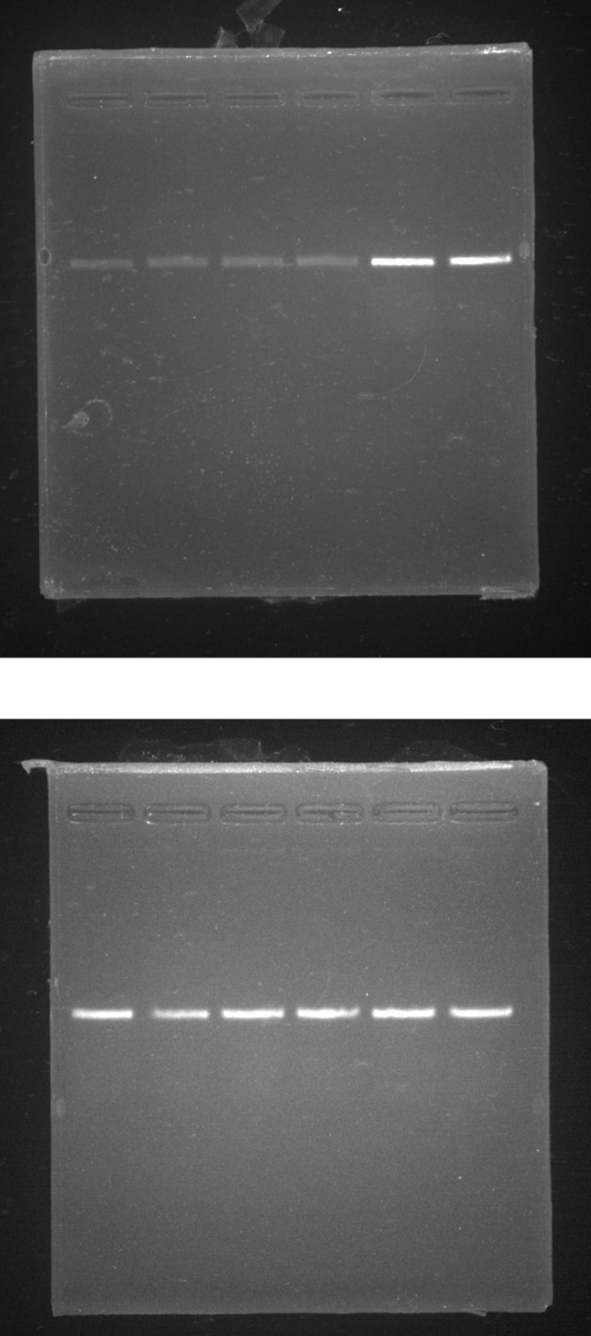

Supplement: Source data 1. [file elife-73347-supp4.zip › Raw_image/Raw image for Figure 4¿Cfigure supplement 1C-1.tif]

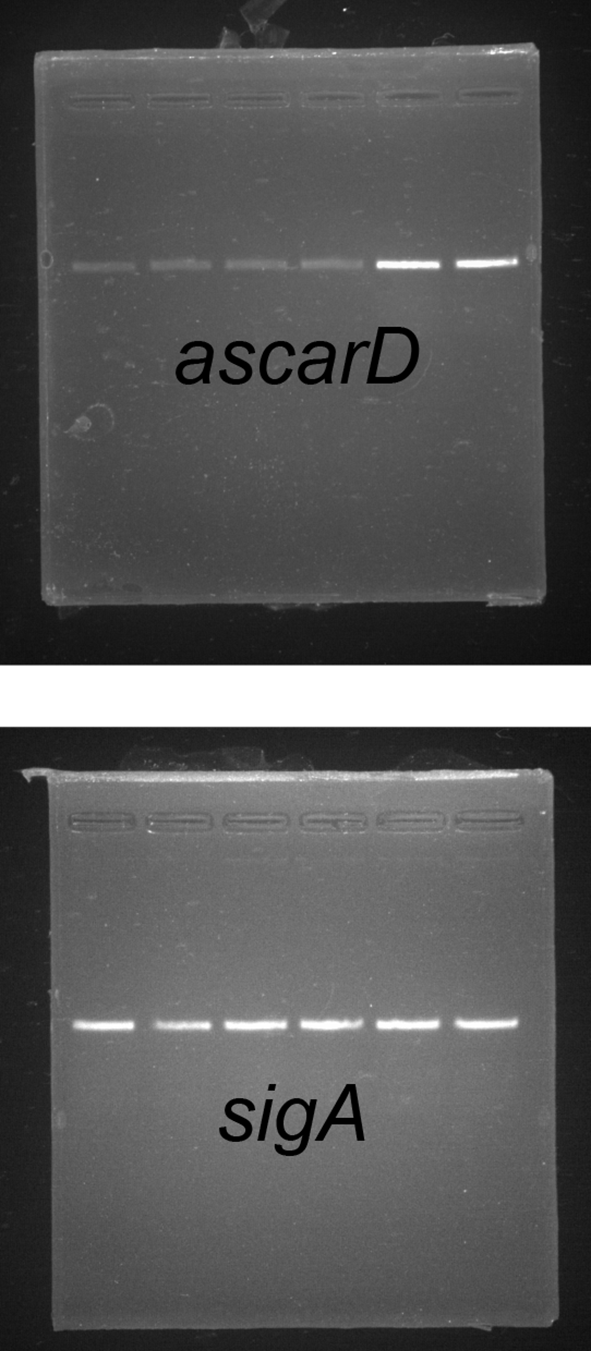

Supplement: Source data 1. [file elife-73347-supp4.zip › Raw_image/Raw image for Figure 4¿Cfigure supplement 1C-2.tif]

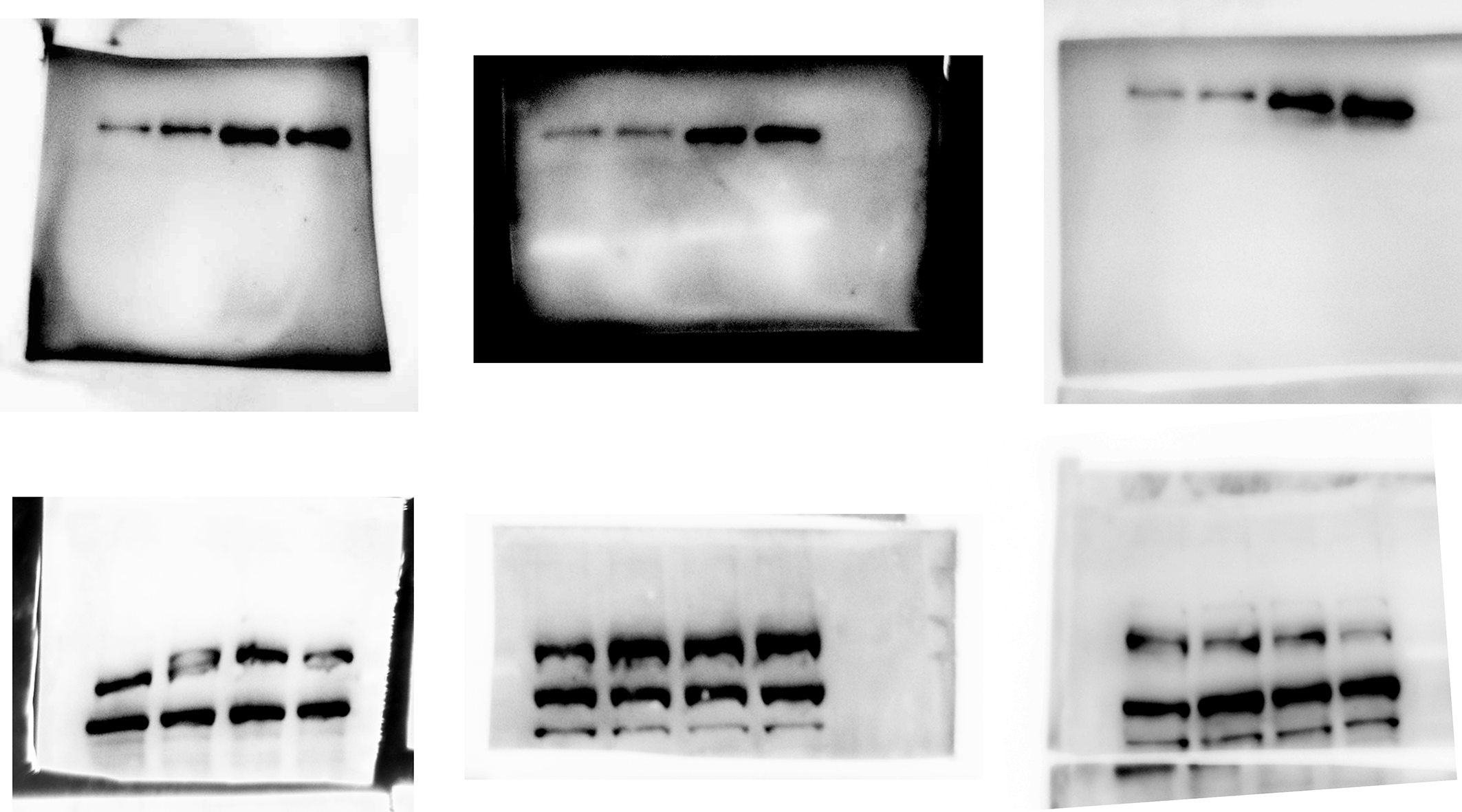

Supplement: Source data 1. [file elife-73347-supp4.zip › Raw_image/Raw image for Figure 6A-1.tif]

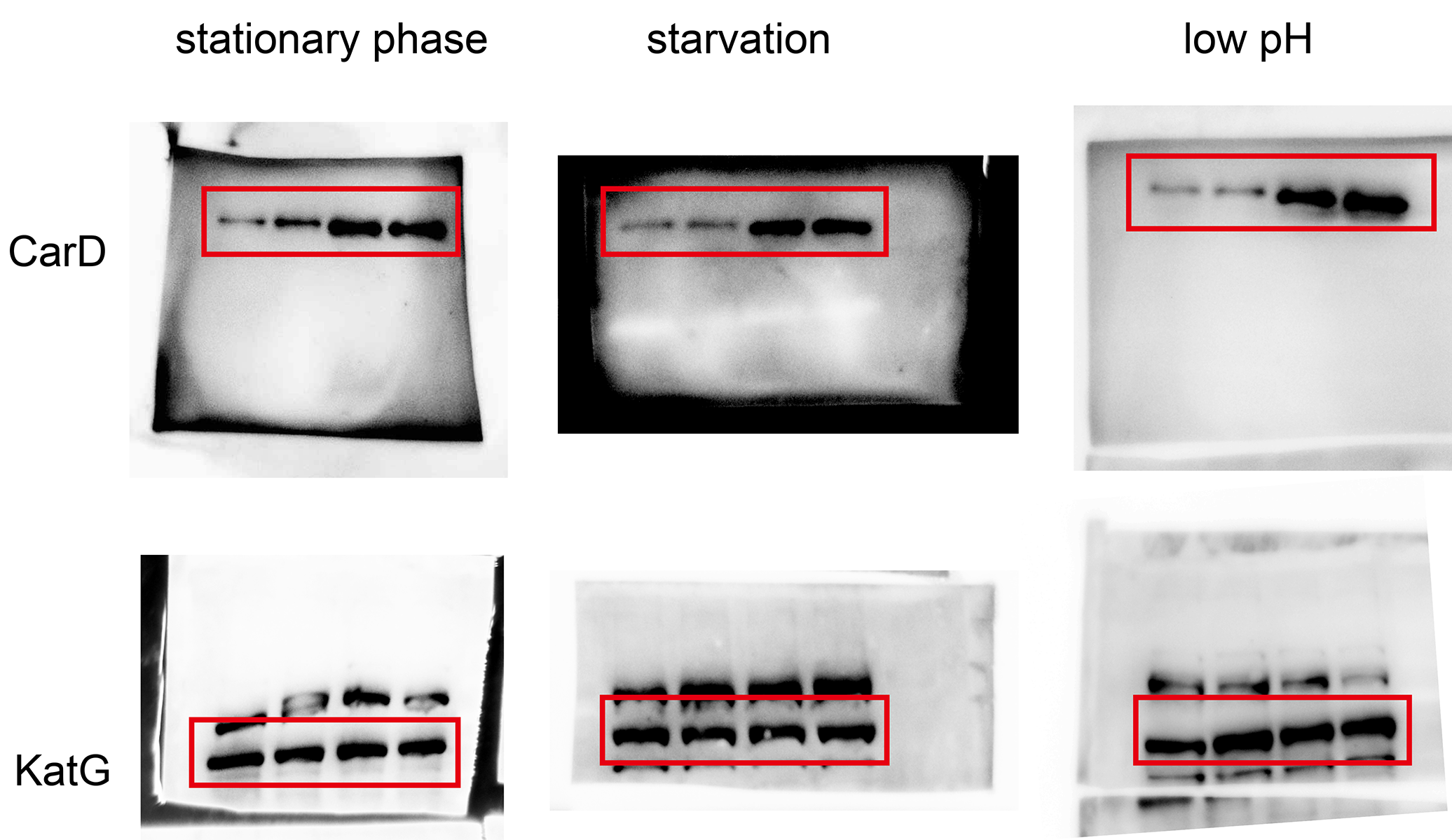

Supplement: Source data 1. [file elife-73347-supp4.zip › Raw_image/Raw image for Figure 6A-2.tif]
